# MRI Lesion Load of Cerebral Small Vessel Disease and Cognitive Impairment in Patients With CADASIL

**DOI:** 10.3389/fneur.2018.00862

**Published:** 2018-10-16

**Authors:** YuZhi Shi, ShaoWu Li, Wei Li, Chen Zhang, LiYing Guo, YunZhu Pan, XueMei Zhou, XinGao Wang, Songtao Niu, XueYing Yu, HeFei Tang, Bin Chen, ZaiQiang Zhang

**Affiliations:** ^1^Department of Neurology, Beijing Tiantan Hospital, Capital Medical University, Beijing, China; ^2^Department of Functional Neuroimaging, Beijing Neurosurgical Institute, Capital Medical University, Beijing, China

**Keywords:** cerebral autosomal-dominant arteriopathy with subcortical infarcts and leukoencephalopathy, cognitive impairment, total small vessel disease score, cerebral atrophy, small vessel disease

## Abstract

**Background and objective:** Cerebral autosomal-dominant arteriopathy with subcortical infarcts and leukoencephalopathy (CADASIL) is the best known and the most common monogenic small vessel disease (SVD). Cognitive impairment is an inevitable feature of CADASIL. Total SVD score and global cortical atrophy (GCA) scale were found to be good predictors of poor cognitive performance in community-dwelling adults. We aimed to estimate the association between the total SVD score, GCA scale and the cognitive performance in patients with CADASIL.

**Methods:** We enrolled 20 genetically confirmed CADASIL patients and 20 controls matched by age, gender, and years of education. All participants underwent cognitive assessments to rate the global cognition and individual domain of executive function, information processing speed, memory, language, and visuospatial function. The total SVD score and GCA scale were rated.

**Results:** The CADASIL group performed worse than the controls on all cognition measures. Neither global cognition nor any separate domain of cognition was significantly different among patients grouped by total SVD score. Negative correlations between the GCA score and cognitive performance were observed. Approximately 40% of the variance was explained by the total GCA score in the domains of executive function, information processing speed, and language. The superficial atrophy score was associated with poor performance in most of the domains of cognition. Adding the superficial atrophy score decreased the prediction power of the deep atrophy score on cognitive impairment alone.

**Conclusions:** The GCA score, not the total SVD score, was significantly associated with poor cognitive performance in patients with CADASIL. Adding the superficial atrophy score attenuated the prediction power of the deep atrophy score on cognitive impairment alone.

## Introduction

Cerebral small vessel disease (SVD) is a major cause of cognitive impairment. Cerebral autosomal-dominant arteriopathy with subcortical infarcts and leukoencephalopathy (CADASIL) is the best known and the most common monogenic SVD and is considered a unique model for the study of sporadic SVD (1). Cognitive impairment is a common symptom of CADASIL.

Neuroimaging signatures of SVD include lacune, deep cerebral microbleeds (CMB), white matter hyperintensities (WMH) of presumed vascular origin, and enlarged perivascular spaces (PVS) (2). These magnetic resonance imaging (MRI) markers alone or in combination was found to be associated with the cognitive impairment in patients with SVD (3–6). The total SVD score was developed to address the combined effects of these MRI findings mentioned above (7). Some studies have shown that the total SVD score is a good predictor of the poor cognitive performance, especially in community-dwelling older adults (8, 9), while different results have been reported in patients with transient ischaemic attack (TIA) or stroke (10).

Cerebral atrophy is another imaging feature of CADASIL and is thought to be resulted from lacunar lesions and tissue microstructure changes (11). Cerebral atrophy was considered playing an important role in cognitive impairment in CADASIL in previous studies (12, 13). The global cortical atrophy (GCA) scale, also known as the Pasquier scale (14), is a cost-effective visual rating scale suited for implementation in clinical practice. The association between the GCA score and cognitive performance in patients with CADASIL is unknown.

In this study, we aimed to investigate the association between the total SVD score, as well as GCA scale, and poor cognitive performance in patients with CADASIL.

## Materials and methods

### Subjects

#### CADASIL patients

CADASIL patients were enrolled at the Department of Neurology, Beijing Tiantan Hospital, between August 2015 and December 2017. Mutation of the NOTCH3 gene was screened in all patients to establish the diagnosis of CADASIL. Patients had a history of stroke within the preceding 3 months were excluded. Patient who could not be able to accomplish the cognition assessment would be removed from the study.

#### Controls

Subjects who were cognitively normal, defined as a Mini-Mental State Examination (MMSE) (15) score >25, and without a history of TIA or stroke were recruited to the group of control. Efforts were made to match the variables of age, gender, and educational years between controls and CADASIL patients. All control subjects underwent a brain MRI scan and no territorial infarctions or other structural brain lesions on brain MRI.

There was no case of alcoholism, drug abuse and any reported physical or mental disease that might impair cognition in all study subjects.

All subjects gave written informed consents. The study was approved by the Medical Ethics Committee of Beijing Tiantan Hospital, Capital Medical University, and was carried out in accordance with the Declaration of Helsinki.

### Cognition assessment

Cognitive performance of all study subjects was measured with a comprehensive cognition assessment.

Global cognition was estimated using Montreal Cognitive Assessment (MoCA) (16), Brief Memory and Executive Test (BMET) (17), and MMSE. Both BMET and MoCA have been validated to be sensitive and effective screening tools for cognitive impairment in patients with CADASIL (18). MMSE was administered as another global cognition measurement to screen the cognitively normal controls.

The executive function domain was measured by HuaShan version of Stroop Color Word Test (SCWT) III (19) and Shape Trail Test (STT) part B (20). The information processing speed domain was measured by the STT part A (20), the SCWT II (19), and the Symbol Digital Modalities Test (SDMT) (21). Memory was measured with the Auditory Verbal Learning Test—Huashan version (AVLT-H) (22). Rey-Osterrieth Complex Figure Copy (RCFT) (23) and 30-item Chinese version Boston Naming Test (BNT) (24) was administered to estimate the visuospatial function and language separately.

### Other measurement

Disability of patients with CADASIL was assessed with the modified Rankin Score (mRS) (25). Depressive symptom was evaluated by the Center for Epidemiological Survey, Depression scale (CES-D) (26).

### MRI lesion load

All subjects were scanned on a 3.0 T MRI scanner. Sequences included axial T1-weighted, axial T2-weighted, and fluid-attenuated inversion recovery (FLAIR). CADASIL patients underwent additional axial diffusion-weighted imaging (DWI) and susceptibility weighted imaging (SWI) sequences scan. The MRI lesion load of patient with CADASIL was evaluated by the total SVD score and the GCA scale. The total SVD score and GCA scale were rated by one experienced vascular neurologist who was blind to all clinical data.

**Figure 1:**
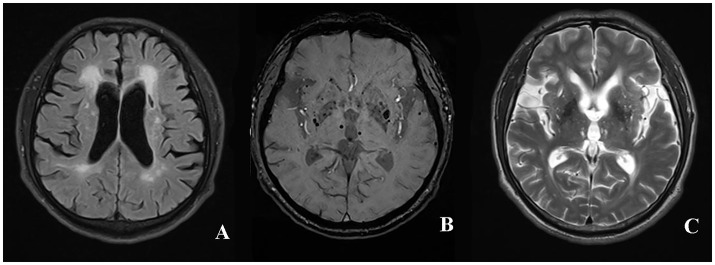
The rating of total SVD score in a patient with CADASIL. The presence of periventricular WMH Fazekas 3 **(A)**, lacune **(B)**, deep CMB **(C)**, and >10 enlarged PVS in unilateral basal ganglia was assigned 1 point, respectively. The total SVD score of the patient was rated 4 for summing up the score of individual SVD imaging marker mentioned above. WMH, white matter hyperintensities; CMB, cerebral microbleed; PVS, perivascular spaces; SVD, small vessel disease; CADASIL, Cerebral Autosomal-Dominant Arteriopathy with Subcortical Infarcts and Leukoencephalopathy.

The total SVD score (7) ranged from 0 to 4 points by adding a point for each of the findings: at least one lacune, at least one deep CMB, moderate to severe WHM [periventricular WMH Fazekas (27) 3 and/or deep WMH Fazekas 2–3] and moderate to severe (>10) PVS in basal ganglia. The rating of the total SVD score in a patient with CADASIL was shown in Figure 1.

**Figure 2:**
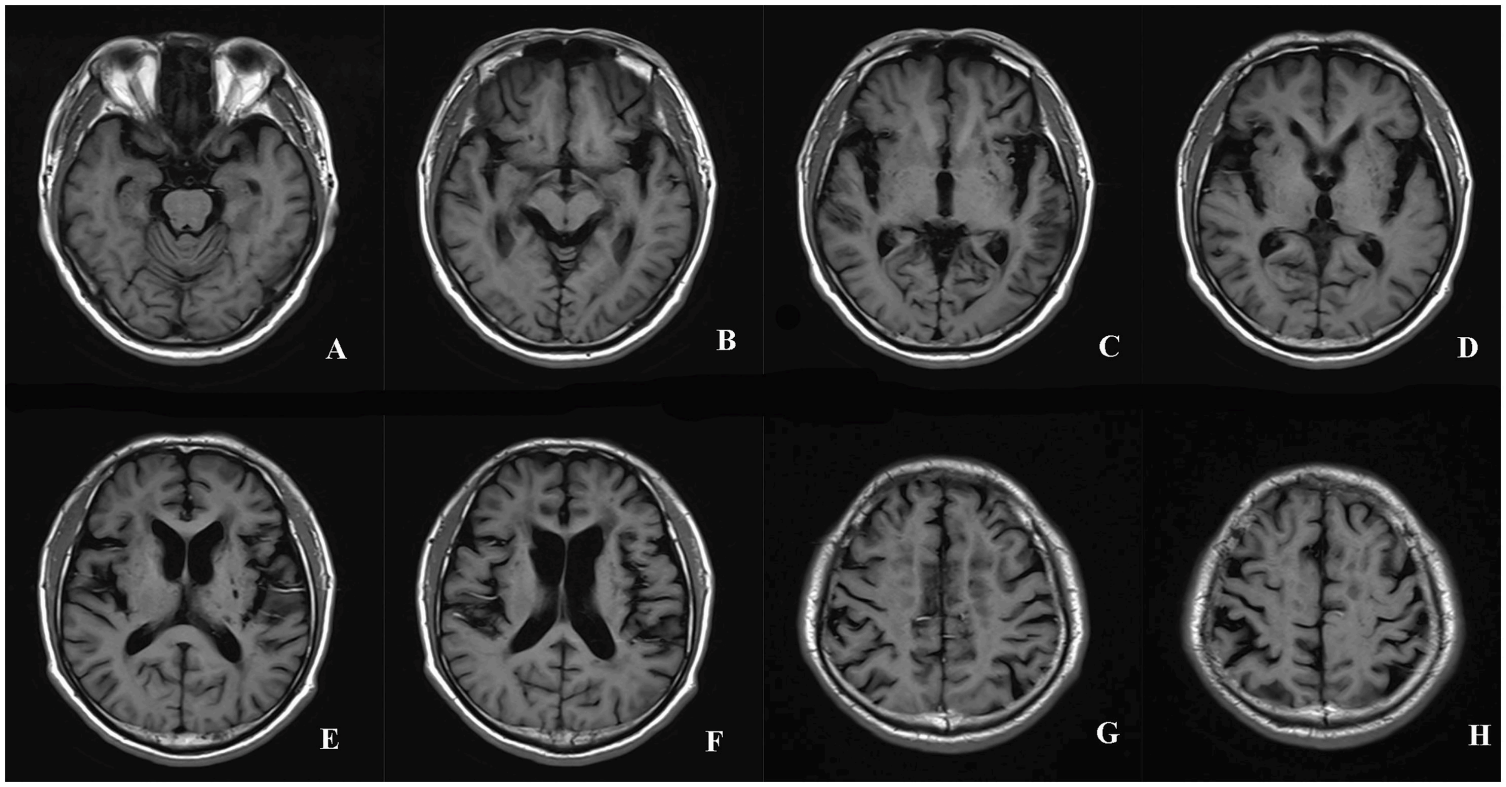
The rating of the GCA scale in a patient with CADASIL. The scores of the sulcal dilation in the regions of bilateral frontal (2+2), parieti-occipital (2+2), and temporal (3+3) were summed to calculate the superficial atrophy score **(A-H)**. The superficial atrophy score of the patient was 14. The dilation of ventricles in bilateral frontal, parieto-occiptial, temporal and the third ventricles was assigned 2+2, 2+2, 2+2, 2 points, respectively **(A-F)**. The deep atrophy score of the patient was 14. Total GCA score was 28. GCA, Global Cortical Atrophy; CADASIL, Cerebral Autosomal-Dominant Arteriopathy with Subcortical Infarcts and Leukoencephalopathy.

The GCA scale (14) was developed by Pasquier to evaluate atrophy degree in 13 brain regions, including the bilateral frontal, parieti-occipital, and temporal sulcal dilation as well as dilation of bilateral frontal, parieto-occiptial, temporal, and the third ventricles. The score for each region can range from 0 to 3 according to the following criteria: 0—normal volume/no ventricular enlargement, 1—opening of sulci/mild ventricular enlargement, 2—volume loss of gyri/moderate ventricular enlargement, 3—“knife blade” atrophy/severe ventricular enlargement. Total GCA score is the sum of the scores of all 13 regions. The sum scores of sulcal dilation and ventricle dilation form the superficial atrophy score and the deep atrophy score, respectively. The rating of the GCA scale in a patient with CADASIL was shown in Figure 2.

### Statistical analysis

Differences in demographics, clinical characteristics and the raw scores of the individual cognition tests between the CADASIL and the control groups were investigated using the independent *t*-test, Mann–Whitney *U*-test and chi-square test.

Individual cognitive test score of the CADASIL patients was converted to standardized *z* score based on the mean value and standard deviation of the controls. To maintain the consistency in the direction of impairment that the lower scores translated into poorer cognitive performance, *z* scores for SCWT and STT were multiplied by −1. The *z* scores of the individual tests were averaged to form the separate cognition domain score.

Differences in cognitive impairment (global cognition and separate domains of cognition) among groups with total SVD score rated to 2, 3, and 4 were investigated by ANOVA.

Simple linear regression analyses were used to calculate the coefficients of determinations (*R*^2^). *R*^2^ denoted the proportion of variance in cognitive impairment that was explained by the GCA score. The association between the score of GCA scale (superficial atrophy, deep atrophy, and total score) and cognition impairment (global cognition and separate domains of cognition) were investigated using multivariable linear regression analyses. The variable of the years of education was entered in the linear regression models as independent variable.

IBM SPSS Statistics 19.0 software was used for statistical analysis. *P* < 0.05 was considered significant for all analyses.

## Results

### Demographic and clinical characteristics

Twenty patients with CADASIL and 20 controls were enrolled in the study. The demographics and medical histories of both groups are shown in Table 1. The CADASIL and control groups did not differ significantly in age (years 49.9 ± 10.0 vs. 49.4 ± 10.7, *P* = 0.840), sex [male, *n*(%), 14 (70.0) vs. 14 (70.0), *P* = 1.000] or years of education (years 10.3 ± 3.5 vs. 10.1 ± 4.2, *P* = 0.880). There were 6 (30%) patients had history of stroke or TIA:4 had ischemic stroke, 1 had TIA and 1 had cerebral hemorrhage. Only one patient who had a history of stroke was disabled (mRS = 3). Compared to the controls, CADASIL patients had a higher prevalence migraine (15 vs. 0%, *P* = 0.036).

**Table 1:** Demographics and clinical characteristics of the participants.

**Demographics and clinical characteristics**	**CADASIL** **(*n* = 20)**	**Controls** **(*n* = 20)**	***P***
**DEMOGRAPHICS**			
Age (years, mean ± SD)	49.9 ± 10.0	49.4 ± 10.7	0.840
Male (%)	14 (70.0)	14 (70.0)	1.000
Educated years, mean ± SD	10.3 ± 3.5	10.1 ± 4.2	0.880
**VASCULAR RISK FACTORS**			
Hypertension, *n* (%)	6 (30.0)	5 (25.0)	0.723
Diabetes mellitus, *n* (%)	2 (10.0)	4 (20.0)	0.372
Hyperlipidaemia, *n* (%)	9 (45.0)	7 (35.0)	0.519
Atrial fibrillation, *n* (%)	0 (0)	0 (0)	/
Cardiovascular disease, *n* (%)	2 (10.0)	0 (0)	0.090
Recurrent stroke/TIA, *n* (%)	6 (30.0)	0 (0)	0.020
Migraine, *n* (%)	3 (15.0)	0 (0)	0.036
Smoking, *n* (%)	7 (35.0)	5 (25.0)	0.490
Drinking, *n* (%)	0 (0)	0 (0)	/

*TIA, transient ischemi stroke; CADASIL, Cerebral autosomal-dominant arteriopathy with subcortical infarcts and leukoencephalopathy. "/" P value can not be calculated*.

The patients with CADASIL scored higher on the CES-D scale compared to the controls, median (Q1–Q3): 6.00 (0.00–14.00) vs. 4.00 (2.00–6.00), *P* < 0.001.

### Cognitive performance

**Figure 3:**
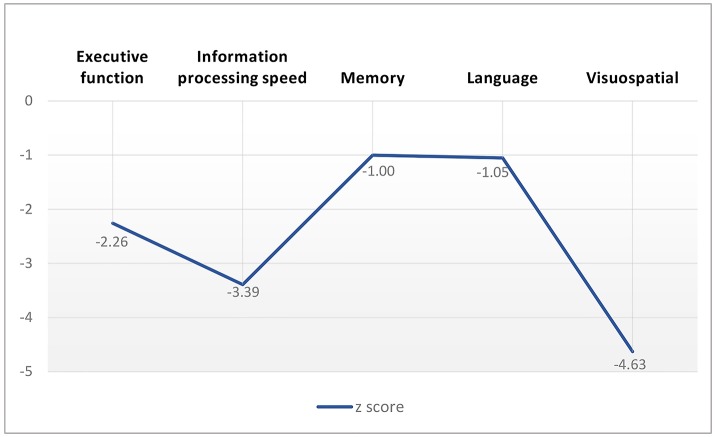
Cognitive performance profile of patients with CADASIL according to *z* score of cognition measures.

The CADASIL group performed worse than the controls on all the cognitive measures (Table 2), with effect sizes (*z* score) range from −4.63 to −1.00 for the domain scores (Figure 3). The domains of executive function, information processing speed and visuospatial function were more severely impaired in the patients with CADASIL than the two other domains.

**Table 2:** Cognitive performances of the CADASIL and the control groups.

**Cognitive performances**	**CADASIL** **(*n* = 20)**	**Control** **(*n* = 20)**	***P***
**GLOBAL COGNITION**			
BMET, mean ± SD	9.15 ± 4.02	14.4 ± 1.66	<0.001
MoCA, mean ± SD	18.45 ± 6.16	25.60 ± 2.62	<0.001
MMSE, mean ± SD	24.65 ± 4.55	29.47 ± 0.84	<0.001
**COGNITION DOMAINS AND MEASURES**			
**Executive function**			
STT part B, mean ± SD	234.88 ± 92.15	115.82 ± 52.04	<0.001
SCWT III, mean ± SD	118.32 ± 58.22	69.58 ± 21.04	<0.001
**Information processing speed**			
STT part A, mean ± SD	133.60 ± 79.86	49.45 ± 15.46	<0.001
SCWT II, mean ± SD	61.67 ± 19.27	39.32 ± 12.94	<0.001
SDMT, mean ± SD	19.60 ± 15.02	41.95 ± 9.62	<0.001
**Memory**			
AVLT-H (Learning), mean ± SD	13.55 ± 5.45	17.60 ± 3.57	0.008
AVLT-H (Short-term delayed recall), mean ± SD	4.30 ± 2.65	6.25 ± 2.12	0.014
AVLT-H (Long-term delayed recall), mean ± SD	3.65 ± 3.11	5.90 ± 2.46	0.016
AVLT-H (Category-cued recall), mean ± SD	3.65 ± 3.08	5.60 ± 2.76	0.042
AVLT-H (Recognition), mean ± SD	8.25 ± 3.95	11.35 ± 3.06	0.009
**Language**			
BNT, mean ± SD	21.75 ± 5.55	24.95 ± 3.05	0.031
**Visuospatial**			
RCFT copy, mean ± SD	22.17 ± 10.89	33.35 ± 2.47	<0.001

*CADASIL, Cerebral Autosomal-Dominant Arteriopathy with Subcortical Infarcts and Leukoencephalopathy; BMET, Brief Memory and Executive Test; MoCA, Montreal Cognitive Assessment; MMSE, Mini-Mental State Examination; STT, Shape Trail Test; SCWT, Stroop Color Word Test; SDMT, Symbol Digital Modalities Test; AVLT-H, Auditory Verbal Learning Test—HuaShan version; BNT, Boston Naming Test; RCFT, Rey-Osterrieth Complex Figure Test*.

### MRI lesion load and cognitive performance in patients with CADASIL

#### SVD score and cognitive performance

In the CADASIL group, 18(90.0%), 17(85.0%), 20(100.0%), and 13(65.0%) of patients present with lacunes, CMB, moderate to severe WMH, and >10 PVS in basal ganglia, respectively. Respectively, 3, 6, and 11 patients were rated to 2, 3, and 4 points according to calculation of the total SVD score, respectively. Neither global cognition nor any separate domain of cognition was significantly different among the patients grouped by the total SVD score (Table 3).

**Table 3:** The association of the total SVD score and cognitive performance in patients with CADASIL.

**Cognitive performances**	**Total SVD score** **= 4 (*n* = 11)**	**Total SVD score** **= 3 (*n* = 6)**	**Total SVD score** **= 2 (*n* = 3)**	***P***
**GLOBAL COGNITION (MEAN** ± **SD)**				
BMET	8.36 ± 4.45	10.33 ± 2.58	9.66 ± 5.50	0.636
MoCA	16.82 ± 6.64	21.33 ± 3.38	18.67 ± 8.50	0.372
***Z*** **SCORES OF THE SEPARATE DOMAINS OF COGNITION (MEAN** ± **SD)**				
Executive function	−2.40 ± 1.94	−2.13 ± 1.49	−2.20 ± 2.32	0.962
Information processing speed	−4.46 ± 3.81	−2.86 ± 1.60	−3.87 ± 3.87	0.645
Memory	−1.18 ± 1.13	−0.86 ± 1.03	−0.62 ± 2.08	0.754
Language	−1.63 ± 1.74	−0.20 ± 1.13	−1.41 ± 2.71	0.138
Visuospatial	−4.14 ± 4.09	−3.35 ± 3.50	−3.25 ± 5.00	0.778

*CADASIL, Cerebral autosomal-dominant arteriopathy with subcortical infarcts and leukoencephalopathy; SVD, small vessel disease; BMET, Brief Memory and Executive Test; MoCA, Montreal Cognitive Assessment*.

#### GCA score and cognitive performance

The mean scores for superficial atrophy, deep atrophy and global atrophy estimated by the GCA scale in patients with CADASIL were 6.30 ± 3.86, 7.9 ± 4.58, and 14.2 ± 8.22, respectively.

Multivariable linear regression analyses showed that the deep atrophy score and total GCA score were significantly associated with the cognitive impairment in global cognition and all domains of cognition after adjustment for the years of education. The superficial atrophy score had negative correlation with cognitive performance in almost all domains of cognition except for the memory. Although the superficial atrophy score associated with the poor cognitive performance, the prediction power of deep atrophy score to cognitive impairment was decreased by adding up superficial atrophy score (Table 4).

**Table 4:** The association of the GCA score and cognitive performance in patients with CADASIL.

**Neuropsychological profile**	**Superficial atrophy** **score, *B* (95%CI)**	**Deep atrophy** **score, *B* (95%CI)**	**Total GCA score, *B* (95%CI)**
**GLOBAL COGNITION**			
BMET	−0.396 (−0.640 to −0.153)	−0.413 (−0.574 to −0.252)	−0.216 (−0.315 to −0.117)
MoCA	−0.356 (−0.605 to −0.108)	−0.345 (−0.534 to −0.156)	−0.186 (−0.295 to −0.077)
**DOMAINS OF COGNITION**			
Executive function	−0.224 (−0.404 to −0.043)	−0.287 (−0.398 to −0.175)	−0.137 (−0.211 to −0.064)
Information processing speed	−0.461 (−0.799 to −0.122)	−0.501 (−0.733 to −0.269)	−0.258 (−0.399 to −0.116)
Memory	−0.109 (−0.253 to −0.034)	−0.141 (−0.248 to −0.034)	−0.068 (−0.131 to −0.005)
Language	−0.277 (−0.478 to −0.076)	−0.269 (−0.422 to −0.115)	−0.145 (−0.233 to −0.056)
Visuospatial	−0.487 (−0.940 to −0.035)	−0.532 (−0.864 to −0.201)	−0.273 (−0.469 to −0.078)

*B, Unstandardized Coefficient; CI, Confidence interval; GCA, Global Cortical Atrophy; CADASIL, Cerebral Autosomal-Dominant Arteriopathy with Subcortical Infarcts and Leukoencephalopathy; BMET, Brief Memory and Executive Test; MoCA, Montreal Cognitive Assessment*.

**Figure 4:**
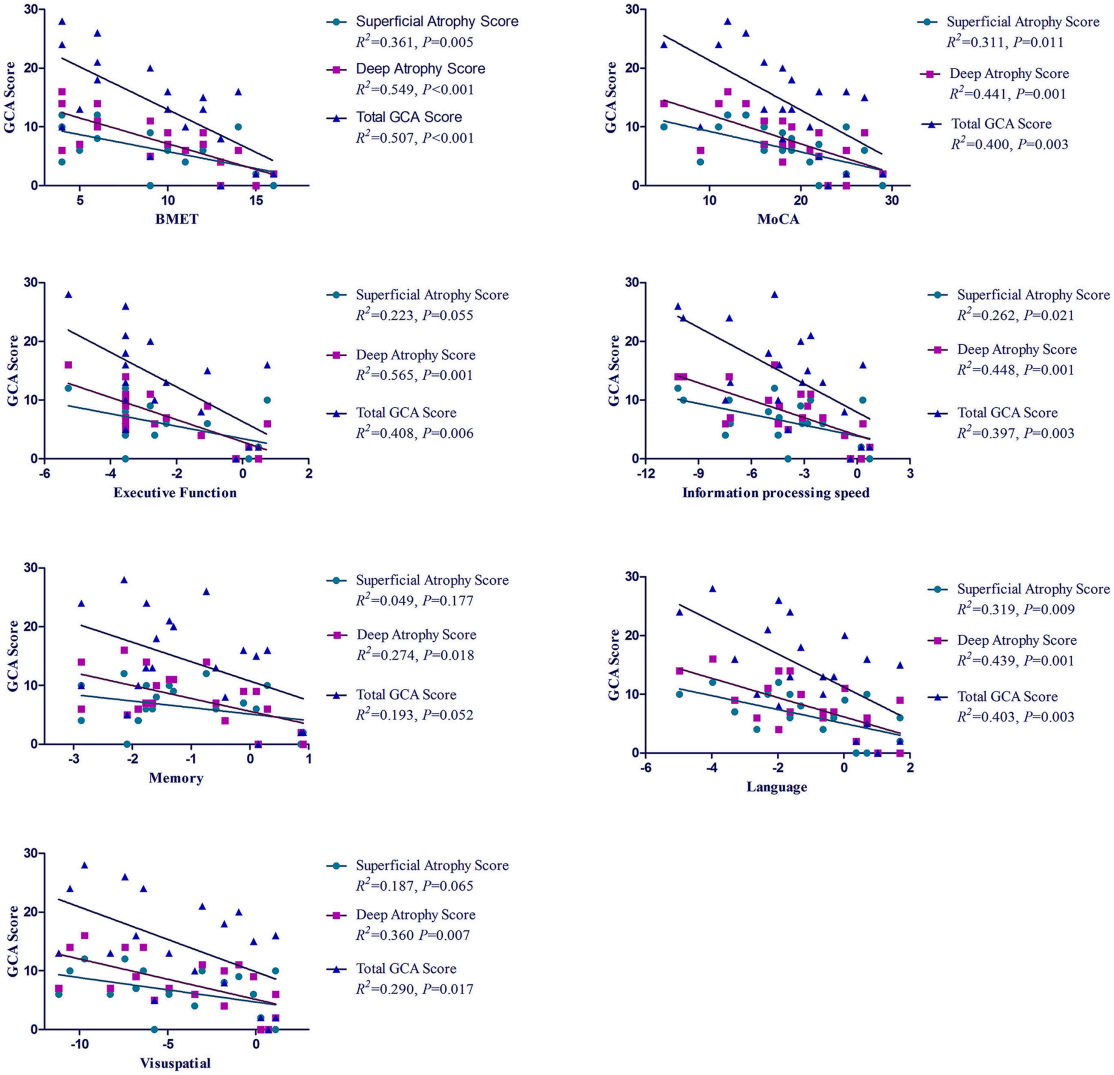
*R*^2^ for the association between GCA score and cognitive impairment in patients with CADASIL. *R*^2^, coefficient of determination; GCA, Global Cortical Atrophy; CADASIL, Cerebral Autosomal-Dominant Arteriopathy with Subcortical Infarcts and Leukoencephalopathy; BMET, Brief Memory and Executive Test; MoCA, Montreal Cognitive Assessment.

Referring to global cognition, the total GCA score explained a larger proportion of the variance in the BMET (50.7%) than in the MoCA (40.0%) with the controls as reference. Approximately 40% of the variance in cognitive impairment could be explained by the total GCA score to the domains of executive function, information processing speed and language. It is noted that the deep atrophy score explained more variance in cognitive impairment than the total GCA score, either in global cognition or in separate domains of cognition, with a higher value of *R*^2^ in all linear regression models (Figure 4).

## Discussion

To the best of our knowledge, this study is the first to examine the association between the total SVD score, GCA scale and cognitive performance in patients with CADASIL. The major finding is that the GCA score, not the total SVD score, is significantly associated with the poor cognitive performance in patients with CADASIL.

Compared with controls, the CADASIL group showed a significant reduction in scores on all measures of global cognition and the separate domains of cognition. The domains of executive function, information processing speed and visuospatial function were much largely impaired in the CADASIL group in our study. According to previous studies, cognitive impairment in CADASIL first involves information processing speed and executive function (1, 28); memory impairment develops later in the disease (28); Visuospatial function deteriorates with age, mainly after age 60 in subjects with CADASIL (29).

The total SVD score was considered to be an independent predictor of poor cognitive performance in community-dwelling older adults, and as much as 1/3 of the MoCA score variance could be explained by the total SVD score (8, 9). In contrast to the results of community-based studies, no significant negative correlations between the total SVD score and cognitive performance were observed in patients with TIA or stroke (10). In the present study, we did not find any significant difference in global cognition or in separate domains of cognition among the CADASIL patients grouped by the total SVD score. The majority of CADASIL patients scored 3 (*n* = 6) or 4 (*n* = 11) on the total SVD score which might limit the statistical power to find differences based on this score. In addition, the total SVD score captures the presence of combined lesions (lacunes, microbleeds, enlarged PVS, WMH) but it does not measure the absolute burden of these lesions. A new tool with quantification the lesions mentioned might be able to describe the lesion burden better than the total SVD score in patients with CADASIL.

Cerebral atrophy is another MRI feature of CADASIL and is not included in the total SVD score. Brain atrophy evaluated by brain parenchymal fraction was found to play an important role in cognitive impairment in CADASIL (12). Similar results were obtained in another study using a different method for the measurement of brain volume (13). We evaluated atrophy using a visual scale and found that GCA score was associated with poor cognitive performance in patients with CADASIL. CADASIL has primarily been defined as a subcortical disease. More and more evidence show that cortical changes might be involved in the cognitive deficits by direct mechanisms or through secondary degeneration (12, 30–33). In the present study we also found that superficial atrophy was negatively associated with poor performance in most of the domains of cognition. However, adding the superficial atrophy score decreased the prediction power of the deep atrophy score on cognitive impairment. We speculate that the superficial atrophy is mainly secondary to subcortical changes and the cognitive deficits are largely attributed to the subcortical origin.

There were a number of limitations to this study. First, the correlations of determination were calculated by multivariable linear regression analyses only adjusted for the years of education due to the small sample size, which may over-estimate the strength of the relationships. Second, depressive disorders and apathy were common symptoms in the patients with CADASIL and patients depressed or apathetic performed worse on cognitive assessments compared to those who were not (34, 35). We did find that the patients with CADASIL had more depressive symptoms than the controls in the present study. Unfortunately, we cannot analyse the association between MRI lesion load and cognitive performance by adjusting for depressive disorders besides the years of education due to the small sample size. However, in patients with SVD, cerebral atrophy was still significantly associated with cognitive decline after adjusting for depressed mood (36). Emotional disorders adversely affect the cognitive performance to some degree, but the foundation behind emotional disorders as well as the cognitive impairment was the pathological changes in brain. Third, we chose the total SVD score and GCA scale to rate the MRI lesion load of patients with CADASIL for their clinical usefulness and conveniences, however, these visual rating scales cannot quantify the MRI lesion load precisely. Fourth, CADASIL patients in the our study had more severe cognition deficits than in some previous studies (28, 29). The generalization of the results might be limited. Despite many limitations mentioned above, the major strengths of this study include the well-matched case-control groups and comprehensive cognition assessment, which included a series of tests in global cognition as well as in five separate cognitive domains.

## Conclusion

No significant association was found between the total SVD score and poor cognitive performance. However, cerebral atrophy was negatively associated with cognitive performance in patients with CADASIL. Although superficial atrophy was significantly associated with cognitive impairment, adding the superficial atrophy score attenuated the predictive power of the deep atrophy score on cognitive impairment alone. The generalization of the results to all CADASIL subjects need to be verified in multi-center studies with large study samples.

## Author contributions

YS, WL, and ZZ: conception and design of the study; XW, SN, XY, HT, BC, LG, XZ, and YP: acquisition of demographic and clinical data; CZ: cognition assessment; SL: MRI lesion load rating; YS: drafting a significant portion of the manuscript.

### Conflict of interest statement

The authors declare that the research was conducted in the absence of any commercial or financial relationships that could be construed as a potential conflict of interest.
